# 6-Benzyl­sulfanyl-9*H*-purine

**DOI:** 10.1107/S1600536809045140

**Published:** 2009-11-04

**Authors:** Ismat Fatima, Munawar Ali Munawar, Misbahul Ain Khan, Sohail Nadeem, Rana Amjad

**Affiliations:** a Institute of Chemistry, University of the Punjab, New Campus, Lahore, Pakistan

## Abstract

The phenyl ring of the title compound, C_12_H_10_N_4_S, a purine derivative, is oriented at a dihedral angle of 76.65 (6)° with respect to the purine ring system. An inter­molecular N—H⋯N hydrogen bonds stabilizes the crystal structure.

## Related literature

For the biological activity of purine derivatives, see: Lepage *et al.* (1964 ▶); Mitsuya & Border (1986 ▶); Ragazzi *et al.* (1989 ▶).
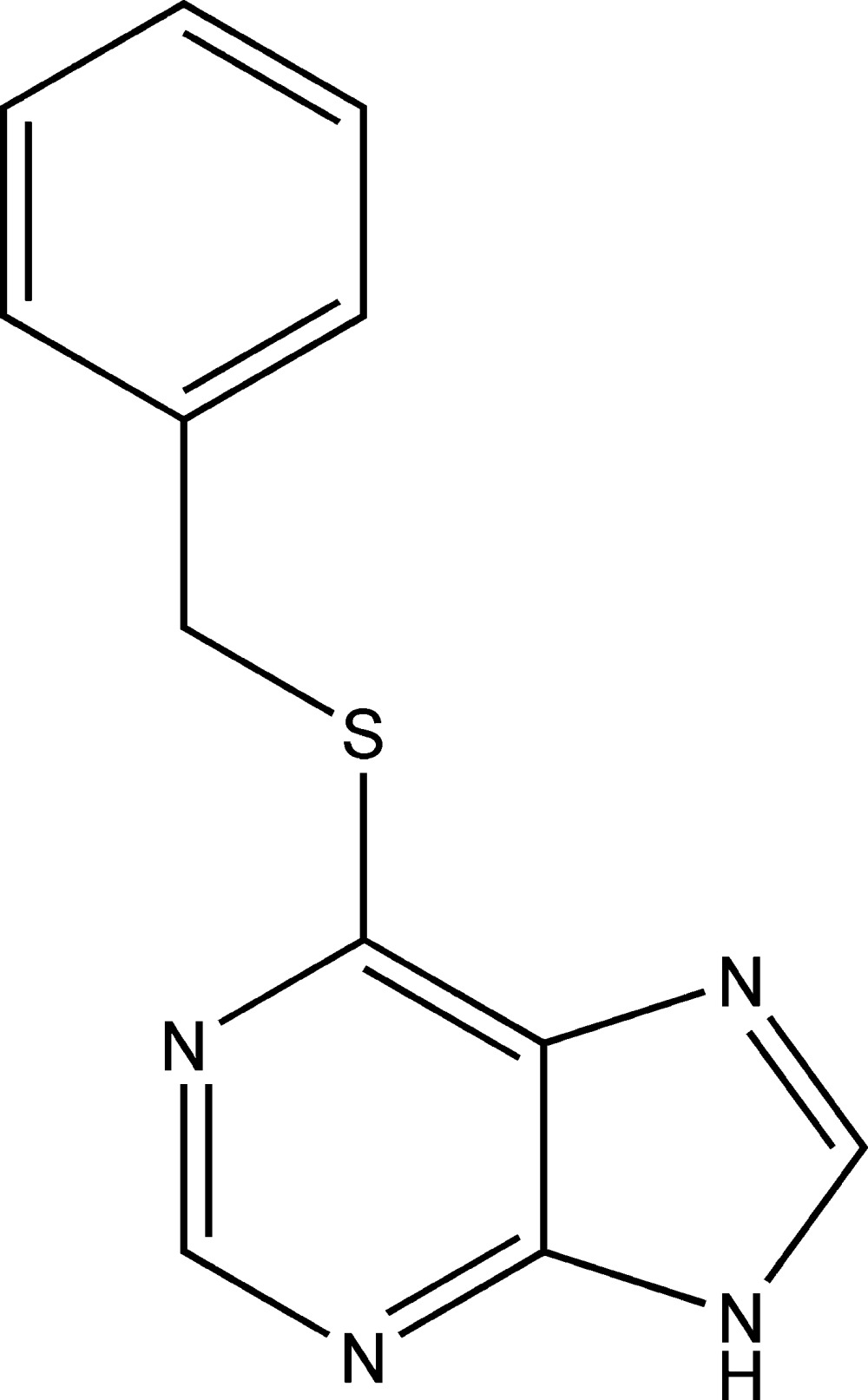


## Experimental

### 

#### Crystal data

C_12_H_10_N_4_S
*M* *_r_* = 242.30
Orthorhombic, 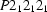

*a* = 5.5717 (3) Å
*b* = 9.4733 (4) Å
*c* = 22.4656 (14) Å
*V* = 1185.79 (11) Å^3^
*Z* = 4
Mo *K*α radiation
μ = 0.26 mm^−1^
*T* = 296 K
0.29 × 0.12 × 0.09 mm

#### Data collection

Bruker Kappa APEXII CCD diffractometer
Absorption correction: multi-scan (*SADABS*; Bruker, 2007 ▶) *T*_min_ = 0.930, *T*_max_ = 0.978
7941 measured reflections
2941 independent reflections
2102 reflections with *I* > 2σ(*I*)
*R*_int_ = 0.032

#### Refinement

*R*[*F* ^2^ > 2σ(*F* ^2^)] = 0.040
*wR*(*F* ^2^) = 0.088
*S* = 0.98
2941 reflections
157 parameters
H atoms treated by a mixture of independent and constrained refinement
Δρ_max_ = 0.17 e Å^−3^
Δρ_min_ = −0.17 e Å^−3^
Absolute structure: Flack (1983 ▶), 1207 Friedel pairs
Flack parameter: −0.09 (8)

### 

Data collection: *APEX2* (Bruker, 2007 ▶); cell refinement: *SAINT* (Bruker, 2007 ▶); data reduction: *SAINT*; program(s) used to solve structure: *SHELXS97* (Sheldrick, 2008 ▶); program(s) used to refine structure: *SHELXL97* (Sheldrick, 2008 ▶); molecular graphics: *ORTEP-3 for Windows* (Farrugia, 1997 ▶) and *PLATON* (Spek, 2009 ▶); software used to prepare material for publication: *WinGX* (Farrugia, 1999 ▶) and *PLATON*.

## Supplementary Material

Crystal structure: contains datablocks I, global. DOI: 10.1107/S1600536809045140/bt5117sup1.cif

Structure factors: contains datablocks I. DOI: 10.1107/S1600536809045140/bt5117Isup2.hkl

Additional supplementary materials:  crystallographic information; 3D view; checkCIF report

## Figures and Tables

**Table 1:** Hydrogen-bond geometry (Å, °)

*D*—H⋯*A*	*D*—H	H⋯*A*	*D*⋯*A*	*D*—H⋯*A*
N3—H3*N*⋯N4^i^	0.894 (19)	1.892 (19)	2.773 (2)	167.9 (18)

Symmetry code: (i) 
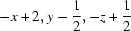
.

**Fig. 1.**
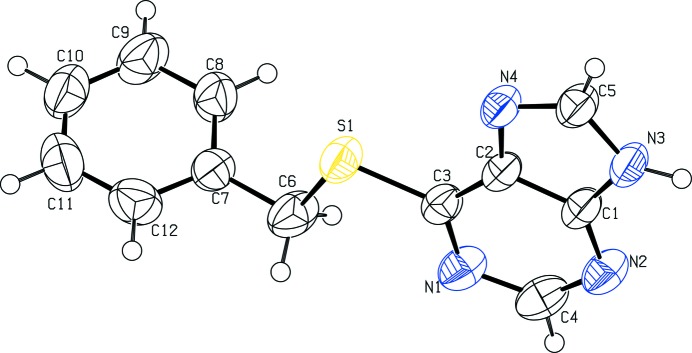
The crystal structure diagram of the title compound duly labeled with 50% probability level of drawn thermal ellipsoids.

**Fig. 2.**
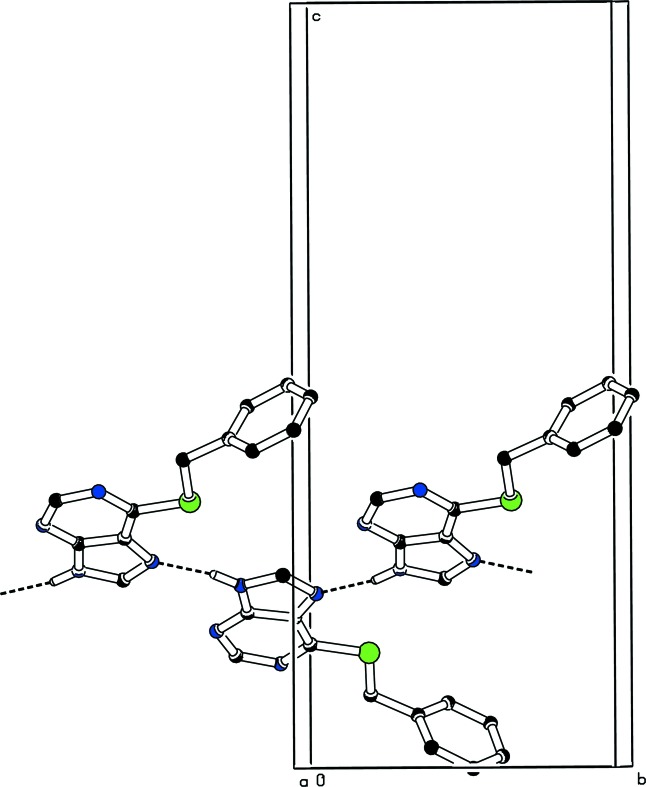
Unit cell diagram showing the intermolecular hydrogen bonding using dashed lines. The hydrogen atoms not involved in hydrogen bonding have been omitted for clarity.

## supplementary crystallographic information

### Comment

The synthesis of purine derivatives has received considerable attention on account of their biological activity especially as antitumor (Lepage *et al.*, 1964), anti HTVL (Mitsuya & Border, 1986) and anti asthmatic (Ragazzi *et al.*, 1989) agents. During our search to find new synthetic antithyroid agents, certain purine derivatives were prepared. 6-(benzylthio)-7*H*-purine was synthesized during such an effort. The compound is now under study for possible antithyroid activity.

The phenyl ring is oriented at adihedral angle of 76.65 (6) ° with respect to purine ring system. An intermolecular N–H···N hydrogen bonds stabilizes the crystal structure.

### Experimental

To a solution of 6-mercaptopurine (0.171 g) 1 mmol in 2 N NaOH (10 ml), benzyl bromide 1 mmol (0.171 g) was added and stirred at room temperature for 30 minutes. The pH of the mixture was adjusted at 5 with glacial acetic acid and the precipitates were collected, washed with water and diethyl ether. The crystals suitable for X-ray diffraction were grown in dichloromethane by slow evaporation at room temperature.

### Refinement

The H-atoms bonded to C were refined geometrically and treated as riding atoms with C_aromatic_—H = 0.93Å and C_methylene_—H = 0.97Å and *U*_iso_(H) = 1.2*U*_eq_(C). The N–H atom was refined at calculated position with N–H=0.894 (19) *U*_iso_(H) = 1.2*U*_eq_ (parent N-atom)

### Crystal data

**Table:**

C_12_H_10_N_4_S	*F*(000) = 504
*M**_r_* = 242.30	*D*_x_ = 1.357 Mg m^−^^3^
Orthorhombic, *P*2_1_2_1_2_1_	Mo *K*α radiation, λ = 0.71073 Å
Hall symbol: P 2ac 2ab	Cell parameters from 2230 reflections
*a* = 5.5717 (3) Å	θ = 2.3–24.8°
*b* = 9.4733 (4) Å	µ = 0.26 mm^−^^1^
*c* = 22.4656 (14) Å	*T* = 296 K
*V* = 1185.79 (11) Å^3^	Needle, red
*Z* = 4	0.29 × 0.12 × 0.09 mm

### Data collection

**Table:**

Bruker Kappa APEXII CCD diffractometer	2941 independent reflections
Radiation source: fine-focus sealed tube	2102 reflections with *I* > 2σ(*I*)
graphite	*R*_int_ = 0.032
φ and ω scans	θ_max_ = 28.3°, θ_min_ = 2.3°
Absorption correction: multi-scan (*SADABS*; Bruker, 2007)	*h* = −7→6
*T*_min_ = 0.930, *T*_max_ = 0.978	*k* = −12→12
7941 measured reflections	*l* = −28→29

### Refinement

**Table:**

Refinement on *F*^2^	Secondary atom site location: difference Fourier map
Least-squares matrix: full	Hydrogen site location: inferred from neighbouring sites
*R*[*F*^2^ > 2σ(*F*^2^)] = 0.040	H atoms treated by a mixture of independent and constrained refinement
*wR*(*F*^2^) = 0.088	*w* = 1/[σ^2^(*F*_o_^2^) + (0.0419*P*)^2^] where *P* = (*F*_o_^2^ + 2*F*_c_^2^)/3
*S* = 0.98	(Δ/σ)_max_ < 0.001
2941 reflections	Δρ_max_ = 0.17 e Å^−^^3^
157 parameters	Δρ_min_ = −0.16 e Å^−^^3^
0 restraints	Absolute structure: Flack (1983), 1207 Friedel pairs
Primary atom site location: structure-invariant direct methods	Flack parameter: −0.09 (8)

### Special details

**Table:**

Geometry. All s.u.'s (except the s.u. in the dihedral angle between two l.s. planes) are estimated using the full covariance matrix. The cell s.u.'s are taken into account individually in the estimation of s.u.'s in distances, angles and torsion angles; correlations between s.u.'s in cell parameters are only used when they are defined by crystal symmetry. An approximate (isotropic) treatment of cell s.u.'s is used for estimating s.u.'s involving l.s. planes.
Refinement. Refinement of *F*^2^ against ALL reflections. The weighted *R*-factor *wR* and goodness of fit *S* are based on *F*^2^, conventional *R*-factors *R* are based on *F*, with *F* set to zero for negative *F*^2^. The threshold expression of *F*^2^ > 2σ(*F*^2^) is used only for calculating *R*-factors(gt) *etc*. and is not relevant to the choice of reflections for refinement. *R*-factors based on *F*^2^ are statistically about twice as large as those based on *F*, and *R*- factors based on ALL data will be even larger.

### Fractional atomic coordinates and isotropic or equivalent isotropic displacement parameters (Å^2^)

**Table:**

	*x*	*y*	*z*	*U*_iso_*/*U*_eq_	
S1	0.47086 (11)	0.20744 (5)	0.15044 (2)	0.05534 (18)	
N1	0.3943 (4)	−0.07206 (16)	0.13660 (7)	0.0553 (5)	
N2	0.6348 (4)	−0.25776 (16)	0.18001 (8)	0.0577 (5)	
N3	0.9619 (4)	−0.16476 (15)	0.23955 (8)	0.0519 (5)	
H3N	1.020 (4)	−0.2456 (19)	0.2541 (8)	0.062*	
N4	0.8929 (3)	0.06585 (15)	0.22833 (7)	0.0488 (4)	
C1	0.7639 (4)	−0.15403 (16)	0.20432 (9)	0.0454 (5)	
C2	0.7219 (4)	−0.01004 (17)	0.19749 (8)	0.0432 (5)	
C3	0.5298 (4)	0.02806 (17)	0.16181 (8)	0.0447 (5)	
C4	0.4558 (4)	−0.2074 (2)	0.14777 (10)	0.0618 (6)	
H4	0.3578	−0.2750	0.1302	0.074*	
C5	1.0294 (4)	−0.03046 (17)	0.25248 (10)	0.0530 (5)	
H5	1.1608	−0.0090	0.2764	0.064*	
C6	0.2345 (4)	0.2002 (2)	0.09542 (10)	0.0662 (6)	
H6A	0.2763	0.1328	0.0646	0.079*	
H6B	0.0862	0.1694	0.1140	0.079*	
C7	0.2003 (4)	0.3442 (2)	0.06836 (9)	0.0500 (5)	
C8	0.3547 (5)	0.3944 (2)	0.02616 (10)	0.0646 (6)	
H8	0.4883	0.3409	0.0158	0.077*	
C9	0.3174 (5)	0.5222 (2)	−0.00144 (11)	0.0741 (8)	
H9	0.4228	0.5532	−0.0307	0.089*	
C10	0.1270 (6)	0.6027 (2)	0.01415 (12)	0.0708 (7)	
H10	0.1011	0.6889	−0.0046	0.085*	
C11	−0.0261 (5)	0.5576 (3)	0.05709 (12)	0.0780 (7)	
H11	−0.1550	0.6139	0.0684	0.094*	
C12	0.0092 (5)	0.4280 (3)	0.08406 (10)	0.0690 (6)	
H12	−0.0976	0.3972	0.1131	0.083*	

### Atomic displacement parameters (Å^2^)

**Table:**

	*U*^11^	*U*^22^	*U*^33^	*U*^12^	*U*^13^	*U*^23^
S1	0.0640 (4)	0.0405 (3)	0.0616 (3)	−0.0021 (3)	−0.0128 (3)	0.0078 (2)
N1	0.0659 (12)	0.0470 (9)	0.0530 (11)	−0.0109 (9)	−0.0074 (9)	0.0019 (8)
N2	0.0708 (12)	0.0373 (8)	0.0650 (12)	−0.0098 (9)	−0.0048 (11)	−0.0040 (8)
N3	0.0602 (12)	0.0309 (7)	0.0647 (11)	0.0005 (8)	−0.0058 (10)	0.0064 (7)
N4	0.0571 (11)	0.0330 (7)	0.0562 (11)	−0.0034 (8)	−0.0085 (9)	0.0020 (7)
C1	0.0582 (14)	0.0303 (9)	0.0478 (11)	−0.0028 (9)	0.0053 (10)	0.0011 (8)
C2	0.0525 (13)	0.0308 (9)	0.0464 (11)	−0.0040 (9)	0.0029 (9)	0.0022 (8)
C3	0.0526 (12)	0.0386 (9)	0.0431 (11)	−0.0025 (9)	0.0044 (10)	0.0049 (8)
C4	0.0758 (16)	0.0467 (11)	0.0627 (13)	−0.0193 (12)	−0.0046 (14)	−0.0087 (11)
C5	0.0587 (14)	0.0378 (9)	0.0625 (13)	−0.0042 (10)	−0.0083 (12)	0.0024 (9)
C6	0.0704 (15)	0.0566 (12)	0.0715 (15)	−0.0128 (12)	−0.0217 (12)	0.0201 (11)
C7	0.0496 (13)	0.0480 (11)	0.0525 (13)	−0.0036 (10)	−0.0095 (10)	0.0048 (10)
C8	0.0681 (16)	0.0550 (12)	0.0705 (15)	0.0111 (11)	0.0186 (13)	0.0048 (12)
C9	0.094 (2)	0.0577 (13)	0.0710 (17)	−0.0028 (15)	0.0181 (15)	0.0168 (12)
C10	0.0819 (19)	0.0491 (12)	0.0814 (18)	0.0038 (13)	−0.0133 (16)	0.0103 (13)
C11	0.0651 (17)	0.0740 (15)	0.0948 (19)	0.0241 (15)	0.0010 (17)	−0.0048 (14)
C12	0.0568 (15)	0.0833 (15)	0.0668 (15)	−0.0018 (15)	0.0091 (13)	0.0148 (12)

### Geometric parameters (Å, °)

**Table:**

S1—C3	1.7495 (17)	C6—C7	1.506 (3)
S1—C6	1.808 (2)	C6—H6A	0.9700
N1—C3	1.338 (2)	C6—H6B	0.9700
N1—C4	1.350 (3)	C7—C8	1.366 (3)
N2—C4	1.322 (3)	C7—C12	1.374 (3)
N2—C1	1.334 (2)	C8—C9	1.376 (3)
N3—C5	1.358 (2)	C8—H8	0.9300
N3—C1	1.362 (3)	C9—C10	1.352 (4)
N3—H3N	0.894 (19)	C9—H9	0.9300
N4—C5	1.306 (2)	C10—C11	1.357 (4)
N4—C2	1.380 (2)	C10—H10	0.9300
C1—C2	1.392 (2)	C11—C12	1.383 (3)
C2—C3	1.385 (3)	C11—H11	0.9300
C4—H4	0.9300	C12—H12	0.9300
C5—H5	0.9300		
			
C3—S1—C6	101.51 (10)	C7—C6—H6A	109.8
C3—N1—C4	116.80 (18)	S1—C6—H6A	109.8
C4—N2—C1	111.39 (16)	C7—C6—H6B	109.8
C5—N3—C1	106.18 (16)	S1—C6—H6B	109.8
C5—N3—H3N	128.6 (13)	H6A—C6—H6B	108.2
C1—N3—H3N	124.8 (14)	C8—C7—C12	117.73 (19)
C5—N4—C2	104.28 (15)	C8—C7—C6	121.1 (2)
N2—C1—N3	128.28 (17)	C12—C7—C6	121.2 (2)
N2—C1—C2	125.9 (2)	C7—C8—C9	121.6 (2)
N3—C1—C2	105.86 (16)	C7—C8—H8	119.2
N4—C2—C3	133.49 (16)	C9—C8—H8	119.2
N4—C2—C1	109.82 (18)	C10—C9—C8	119.9 (2)
C3—C2—C1	116.67 (18)	C10—C9—H9	120.1
N1—C3—C2	119.75 (16)	C8—C9—H9	120.1
N1—C3—S1	121.40 (16)	C9—C10—C11	120.0 (2)
C2—C3—S1	118.85 (14)	C9—C10—H10	120.0
N2—C4—N1	129.52 (19)	C11—C10—H10	120.0
N2—C4—H4	115.2	C10—C11—C12	120.1 (2)
N1—C4—H4	115.2	C10—C11—H11	120.0
N4—C5—N3	113.9 (2)	C12—C11—H11	120.0
N4—C5—H5	123.1	C7—C12—C11	120.7 (2)
N3—C5—H5	123.1	C7—C12—H12	119.7
C7—C6—S1	109.51 (15)	C11—C12—H12	119.7
			
C4—N2—C1—N3	−179.25 (19)	C6—S1—C3—C2	173.43 (16)
C4—N2—C1—C2	−0.2 (3)	C1—N2—C4—N1	1.0 (3)
C5—N3—C1—N2	179.4 (2)	C3—N1—C4—N2	−0.8 (3)
C5—N3—C1—C2	0.2 (2)	C2—N4—C5—N3	0.4 (2)
C5—N4—C2—C3	−178.5 (2)	C1—N3—C5—N4	−0.4 (2)
C5—N4—C2—C1	−0.2 (2)	C3—S1—C6—C7	−165.98 (17)
N2—C1—C2—N4	−179.20 (19)	S1—C6—C7—C8	77.9 (2)
N3—C1—C2—N4	0.0 (2)	S1—C6—C7—C12	−103.9 (2)
N2—C1—C2—C3	−0.6 (3)	C12—C7—C8—C9	−2.2 (4)
N3—C1—C2—C3	178.62 (17)	C6—C7—C8—C9	176.1 (2)
C4—N1—C3—C2	−0.2 (3)	C7—C8—C9—C10	1.6 (4)
C4—N1—C3—S1	179.49 (15)	C8—C9—C10—C11	0.3 (4)
N4—C2—C3—N1	179.0 (2)	C9—C10—C11—C12	−1.4 (4)
C1—C2—C3—N1	0.8 (3)	C8—C7—C12—C11	1.0 (4)
N4—C2—C3—S1	−0.7 (3)	C6—C7—C12—C11	−177.3 (2)
C1—C2—C3—S1	−178.90 (15)	C10—C11—C12—C7	0.8 (4)
C6—S1—C3—N1	−6.23 (19)		

### Hydrogen-bond geometry (Å, °)

**Table:**

*D*—H···*A*	*D*—H	H···*A*	*D*···*A*	*D*—H···*A*
N3—H3N···N4^i^	0.894 (19)	1.892 (19)	2.773 (2)	167.9 (18)

Symmetry codes: (i) −*x*+2, *y*−1/2, −*z*+1/2.

## References

[bb1] Bruker (2007). *APEX2*, *SAINT* and *SADABS*. Bruker AXS Inc., Madison, Wisconsin, USA.

[bb2] Farrugia, L. J. (1997). *J. Appl. Cryst.* **30**, 565.

[bb3] Farrugia, L. J. (1999). *J. Appl. Cryst.* **32**, 837–838.

[bb4] Flack, H. D. (1983). *Acta Cryst.* A**39**, 876–881.

[bb5] Lepage, G. A., Junga, J. G. & Bowman, B. (1964). *Cancer Res.* **24**, 835–840. 14190549

[bb6] Mitsuya, H. & Border, S. (1986). *Proc. Natl. Acad. Sci. USA*, **83** 1911–1915. 10.1073/pnas.83.6.1911 PMC323194 3006077

[bb7] Ragazzi, E., Froldi, G., Santi-Sonein, E., Borea, P. A. & Fassina, G. (1989). *Pharmacol Res.* **21**,707–717. 10.1016/1043-6618(89)90230-2 2560547

[bb8] Sheldrick, G. M. (2008). *Acta Cryst.* A**64**, 112–122. 10.1107/S0108767307043930 18156677

[bb9] Spek, A. L. (2009). *Acta Cryst.* D**65**, 148–155. 10.1107/S090744490804362X PMC2631630 19171970

